# Honokiol attenuate the arsenic trioxide‐induced cardiotoxicity by reducing the myocardial apoptosis

**DOI:** 10.1002/prp2.914

**Published:** 2022-02-16

**Authors:** An‐Liang Huang, Fan Yang, Ping Cheng, Dian‐ying Liao, Li Zhou, Xing‐Li Ji, Dou‐Dou Peng, Li Zhang, Ting‐Ting Cheng, Li Ma, Xian‐Gen Xia

**Affiliations:** ^1^ Department of Pathology Chengdu Fifth People’s Hospital Chengdu Sichuan People’s Republic of China; ^2^ Department of Pathology The Fifth Affiliated People's Hospital of Chengdu University of Traditional Chinese Medicine Chengdu Sichuan People’s Republic of China; ^3^ State Key Lab of Biotherapy West China Hospital Sichuan University Chengdu Sichuan People’s Republic of China; ^4^ Department of Pathology West China Hospital Chengdu Sichuan People’s Republic of China

**Keywords:** apoptosis, arsenic trioxide, cardiac remodeling, Honokiol, ROS

## Abstract

Despite advantages of arsenic trioxide (ATO) in oncological practice, its clinical applications have been hampered by severe cardiotoxicity. The general mechanism of ATO‐induced cardiotoxicity has been attributed to its damage to mitochondria, resulting in cardiac remodeling. Honokiol (HKL) is a naturally occurring compound derived from *Magnolia* bark. Previous studies have demonstrated that HKL exerts cardio‐protective effects on ischemia/reperfusion (I/R) or chemical‐induced cardiotoxicity by counteracting the toxic effects on mitochondria. The present study was conducted to investigate whether HKL pretreatment protects against ATO‐induced cardiac oxidative damage and cell death. For the in vitro study, we evaluated the effects of ATO and/or Honokiol on reactive oxygen species (ROS) production and apoptosis induction in primary cultured cardiomyocytes; for the in vivo study, BALB/c mice were administrated with ATO and/or HKL for a period of 4 weeks, myocardial apoptosis, cardiac function, and cardiac remodeling (cardiac hypertrophy and cardiac fibrosis) were assessed at the end of administration. Our results demonstrated Honokiol pretreatment alleviated the ATO‐induced boost in ROS concentration and the following apoptosis induction in primary cultured cardiomyocytes. In the mouse model, Honokiol pretreatment ameliorated ATO‐induced myocardial apoptosis, cardiac dysfunction, and cardiac remodeling. Collectively, these results indicated that Honokiol provide a protection against ATO‐induced cardiotoxicity by reducing mitochondrial damage. In addition, given that Honokiol has shown considerable suppressive effects on leukemia cells, our data also imply that ATO and Honokiol combination may possibly be a superior avenue in leukemia therapy.

## Graphical abstract

In this work, we evaluated protective role of Honokiol, an active compound purified from the bark of *Magnolia Officinalis*, on cardiotoxicity induced by arsenic trioxide (ATO) treatment. The present study conducted has shown that Honokiol pretreatment protects against ATO‐induced cardiac oxidative stress and cell death.

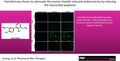

**Abbreviations**
APL: acute promyelocytic leukemia
ATO: arsenic trioxide
ATRA: all‐trans‐retinoic acid
EF: ejection fraction
FS: fractional shortening
HKL: Honokiol
I/R: ischemia/reperfusion
ROS: reactive oxygen species

## 1 INTRODUCTION

Arsenic and its derivatives have been applied in traditional Chinese medicine for thousands of years. Arsenic trioxide (ATO) is being selected as a first‐ and second‐line therapy for the treatment of both newly diagnosed and all‐trans‐retinoic acid (ATRA)‐refractory acute promyelocytic leukemia (APL) patients.^1^ Besides, an overwhelming number of preclinical studies have demonstrated that ATO has the ability to induce apoptosis and inhibit tumor cell growth in a wide variety of solid tumors, including acute myeloid leukemia,^2^, ^3^ multiple myeloma,^4^ head and neck,^5^ and glioblastoma.^6^ Despite its benefits toward cancer treatment, the clinical utility of ATO has been strongly limited due to its cardiotoxic effects, such as QT interval prolongation and torsades de pointes, which mainly manifest as arrhythmia, and even sudden cardiac death.^7^, ^8^

The heart is an organ that consumes massive energy supply. In order to maintain normal contractile coupling, cardiomyocytes rely on continuous oxygen supply for cellular respiration in terms of oxidative phosphorylation, producing enough ATP to meet their significant energetic requirement.^9^, ^10^ Mitochondria is the primary organelle for cellular energy production in form of ATP. In addition to maintaining the normal physiological function of cardiomyocytes, the mitochondrial energy production also participates in the regulation of metabolism of intracellular Ca^2+^.^11^ During the course of cardiomyopathy, the alteration of mitochondrial structure and function lead to the destruction of mitochondrial homeostasis, resulting in disruption of mitochondrial membrane integrity, elevation of intracellular Ca^2+^ concentration, and increased generation of mitochondrial‐derived reactive oxygen species (ROS).^12^, ^13^ These events interact with each other and play an essential role in controlling the pathological development and cell death of cardiomyocytes. In recent years, the molecular mechanism underlying the cytotoxic effect of ATO on cardiomyocytes has been gradually elucidated. In particular, the role of mitochondria dysfunction in myocardial injury has been widely concerned.^14^ Accumulating evidence has demonstrated that arsenic trioxide contribute to cardiovascular toxicity by disrupting mitochondrial function via a series of mechanism, especially by promoting the opening of mitochondrial PTPs and damaging the component of electron transport system, leading to decreased ATP generation, increased ROS production, and subsequent initiation of apoptosis or necrosis.^15^ Therefore, strategies that attenuate mitochondrial injury and improve the function of mitochondrial oxidative phosphorylation may represent a promising therapeutic approach for preventing cardiac toxicity induced by arsenic trioxide.

Honokiol, an active compound purified from the bark of *Magnolia Officinalis*, has drawn much attention for its antitumor, anti‐inflammatory, and antioxidative properties. In published literature, earlier study found that Honokiol was an effective antioxidant in inhibiting lipid peroxidation in rat mitochondria.^16^ In addition, Honokiol has been demonstrated to exert pharmacological effect on myocardial ischemia/reperfusion (I/R) injury, which is associated with the attenuation of oxidative stress by inhibition of NF‐κB pathway.^17^ In recent years, a series of studies focused on the biological action and mechanisms of Honokiol for protecting heart from Dox‐cardiotoxicity in mice models, and these results proposed that Honokiol could protect cardiomyocytes from doxorubicin‐induced cell death via improving mitochondrial function.^18^, ^19^ Based on aforementioned studies, we hypothesized that combination of Honokiol with As_2_O_3_ would be a novel recipe with the potential for mitigating the ATO‐induced cardiotoxicity in association with mitochondria protection. In current study, we investigated the protective effect of Honokiol against ATO‐induced cardiotoxicity both in primary cultured cardiomyocytes and in mice models. Furthermore, we also elucidate its underlying molecular mechanisms regarding antagonism against mitochondrial‐derived ROS and prevention from cardiomyocytes apoptosis.

## 2 MATERIALS AND METHODS

### 2.1 Reagents

Honokiol with purity >98% (sigma) was dissolved in corn oil. Arsenic trioxide was obtained from Sigma‐Aldrich. Arsenic trioxide was dissolved in 1.65 M NaOH at 5 × 10^−2^ M as a stock solution. Monoclonal GAPDH and anti‐SIRT3 were purchased from Cell Signaling Technology. All other antibodies were obtained from Santa Cruz Biotechnology. The protein assay kit was purchased from Bio‐Rad Laboratories. All the chemicals employed in the study were of analytically pure and of culture grade.

### 2.2 Adult mouse cardiomyocytes isolation and culturing

Primary cultures of cardiomyocytes were prepared from adult mouse hearts as described.^20^ Ethical approval for the present study was provided by the Ethics Committee of State Key Laboratory of Biotherapy, Sichuan University. In brief, adult C57BL/6 male mouse (8–10 weeks old) was anesthetized with isoflurane. After opening the chest, the heart was immediately flushed by injection of 7 ml EDTA buffer into the left ventricle, then transferred to a fresh dish. The EDTA buffer, perfusion buffer, and collagenase buffer were consecutively injected into the left ventricle to digest the heart tissue. The heart was gently pulled into pieces using a sterile forceps and pipetted until the majority of myocytes were completely dissociated. Then the stop buffer was added. The cell suspension was filtered through a 100 μm nylon mesh and placed in the conical tube upright to allow the cells to settle by gravity. The cells underwent four sequential rounds of gravity settling using three intermediate calcium buffers to gradually restore calcium concentration to physiological levels. After removing the supernatant at last round, cells were collected and plated in laminin‐coated culture plates. The majority of cells exhibit rod shape with crisp striations as opposed to dead rounded cells. Twenty‐four hours after plating, cardiomyocytes cultures were treated with corn oil, HKL (10 μM), ATO (4 μM), HKL (6 h before ATO was added) + ATO, respectively. Twenty‐four hours after treatment, cells were harvested for ROS level determination, apoptosis, caspase‐3 activation assay, and MAPK pathway signaling.

### 2.3 Animal study

C57BL/6 male mice 8 to 10 weeks old, weighing 22–25 g, were obtained from Beijing WeitongLihua Biological Technology Co., Ltd. Mice were maintained in a specific pathogen‐free facility with 12‐h light‐dark cycles. They were observed for signs of tumor growth, activity, feeding, under the guidelines of the Institutional Animal Care and Use Committee. Thirty‐two of these mice were randomly assigned into the following four groups (*n* = 8): sham versus arsenic trioxide treated mice with or without HKL, and were injected intraperitoneally (i.p.) with arsenic trioxide at dose of 4 mg/kg once every 4 days for 4 weeks (a total of seven injection). HKL treatment (0.2 mg/kg, i.p.) started 6 h before the start of ATO treatment. In control mice, vehicle (corn oil) was used. After echocardiographic assessment, all animals were sacrificed, and hearts were collected and grinded in liquid nitrogen followed by storage at −80°C for protein assay. In the meantime, hearts were homogenized in Tris‐HCl buffer for reactive oxygen species measurement. Hearts were also fixed with formalin for histological analysis.

### 2.4 Isolation of mitochondria from mouse heart

The freshly harvested heart was dissected and dissociated into small pieces (~200 mg) using surgical scissors. After clearing from blood vessels, the tissues were homogenized with a Dounce homogenizer in chilled isolation buffer (225 mM D‐mannitol, 75 mM sucrose, 5 mM KH_2_PO_4_, and 0.5 mM EDTA, pH 7.4) in ice‐cold bath. The homogenate was centrifuged at 200*g* for 5 min at 4°C. After collecting the supernatant, the pellet was re‐suspended in isolation buffer and re‐centrifuged at 1000*g* for 10 min at 4°C; the resulting supernatant containing mitochondria were centrifuged at 8000*g* for 10 min at 4°C. The final mitochondrial pellet after this centrifugation was re‐suspended in chilled respiration‐compatible buffer (225 mM D‐mannitol, 75 mM sucrose, 5 mM KH_2_PO_4_, and 10 mM Tris‐HCl, pH 7.0). The purity and integrity of mitochondria were tested by lactate dehydrogenase and succinate dehydrogenase assays.

### 2.5 Measurement of oxidative stress levels

The oxidation‐sensitive fluorescent probe 5‐(and‐6)‐carboxy 2’, 7’‐dichlorodihydrofluorescein diacetate (carboxy‐DCFDA) was used to measure the redox state of a cell and heart tissue lysates. It is a cell permeable and non‐fluorescent precursor of DCF that is highly fluorescent and can be measured by increase in fluorescence at 530 nm. Twenty‐four hours after treatment, Cells were incubated with carboxy‐H2DCFDA at a final concentration of 1 μM in regular culture medium for 30 min in the dark, and washed twice with PBS, the fluorescence density (arbitrary units (AU)) was acquired using fluorescence plate reader, image of DCF staining was also taken by microscope. For in vivo study, the redox state in heart tissue lysates were measured by the same procedure. To measure mitochondrial superoxide, the fluorescent probe MitoSOX™ Red was used according to the manufacturer's protocol. As mentioned above, cells were washed once with DMEM and incubated with 5 μM MitoSOX™ Red for 10 min and the stained with Hoechst for 20 min at 37°C, followed by fixing in 4% paraformaldehyde. Images were taken on a Leica TCS SPE confocal microscope. The mean values of the whole cell fluorescence of MitoSOX™ Red were obtained with Image J software. To detect the hydrogen peroxide levels, freshly isolated mitochondria from heart tissues were incubated with Amplex Red reagent (10‐acetyl‐3, 7‐dihydroxyphenoxazine), according to the manufacturer's instructions (Amplex Red hydrogen peroxide/peroxidase assay kit, ThermoFisher Scientific). Briefly, 50 μl of the sample in each group, a positive control (10 μM of diluted 20 mM H_2_O_2_ working solution in 1× Reaction Buffer) and negative control (1× reaction buffer without H_2_O_2_) were loaded into individual wells of a micro‐plate. 50 μl of a working solution of 100 μM Amplex Red reagent and 0.2 U/ml HRP was added to each micro‐plate well. The micro‐plate was incubated at room temperature for 30 min (protected from light), and the absorbance was measured at 560 nm in a 96‐well microplate reader.

### 2.6 Apoptosis and caspase‐3 activation assay

The ATO‐induced cardiomyocytes apoptosis was assessed by using Hoechst 33342/annexin V staining. The cardiomyocytes were cultured in 6‐well plates for 24 h. After designated treatment, the cells were washed with annexin V binding buffer, incubated with annexin V‐FITC for 15 min at room temperature, and then counterstained with Hoechst 33342 for nuclei, after washing twice with PBS, the images of apoptotic cells were taken on a fluorescence microscopy. Caspase‐3 activation was measured by caspase 3 colorimetric assay kit (CASP‐3‐C, sigma) which is based on the hydrolysis of the peptide substrate acetyl‐Asp‐Glu‐Val‐Asp p‐nitroanilide (Ac‐DEVD‐pNa) by caspases‐3, resulting in the release p‐nitroaniline (pNA) moiety. Absorbance of pNA in each designated group as mentioned above is measured at 405 nm wavelength in ELISA reader using 96 well plate microassay method, according to the manufacture's instruction.

### 2.7 Western blot analysis

Briefly, 1×105 primary cultured cardiomyocytes were lysed in lysis buffer. The frozen cardiac tissues were lysed in a RIPA buffer, cells were centrifuged at 12 500 rpm for 30 min. The protein concentration of the supernatant was determined by the Bio‐Rad protein assay kit, and whole‐cell lysates after denaturing were separated by 10% SDS–PAGE. Gels were electroblotted onto a poly (vinylidene difluoride) membrane. The membrane blots were blocked at 4°C in 5% nonfat dry milk overnight and incubated with each antibody at a recommended dilution for 8 h at 37°C. Followed by rinsing in solution with 10 mM Tris‐HCl pH 7.5, 100 mM NaCl, and 0.1% Tween‐20 (TBS‐T), the gels were incubated in horseradish peroxidase‐conjugated secondary antibodies at a dilution of 1:10 000. The immunoreactive bands were detected by enhanced chemilluminescence (Amersham Corp.) followed by autoradiography. Equal loading was confirmed by detection of GAPDH.

### 2.8 Real time RT‐PCR

Total RNA was isolated from in vitro cardiomyocytes 24 h after treatment and liquid nitrogen snap‐frozen heart tissue, respectively, using TRIzol reagent (Invitrogen) and treated for 45 min at 37°C with RQ1 DNase (Promega). RNA was reverse transcribed using oligo (dT) primers with the Advantage RT‐for‐PCR kit (Roche), according to the manufacturer's instructions. The Real‐time PCR was done in an ABI Prism 7000 Sequence Detection System (Applied Biosystems) using SYBR Green PCR Master mix (Applied Biosystems) and the thermocycler conditions recommended by the manufacturer. GAPDH was used as reference genes to normalize for differences in the amount of total RNA in each sample. Melting curve analysis showed a single sharp peak with the expected Tm for all samples. mRNA relative quantities were obtained using the 2^−△△^ *^C^* ^t^ method. Primer pairs for amplification of indicated genes are: Anp‐mouse: 5’‐ACCTGCTAGACCAC CTGGAG‐3’ (forward), 5’‐CCTTGGCTG TTATCTTCGGTACCGG‐3’ (reverse). Bnp‐mouse: 5’‐GAGGTCACTCCTATCC TCTGG‐3’ (forward), 5’‐GCCATTTCC TCCGACTTTTCTC‐3’ (reverse). β‐Mhc–mouse: 5’‐CCGAGTCCCAGG TCAA CAA‐3’ (forward), 5’‐CTTCACGGGCACCCTTGGA‐3’ (reverse). Collagen I‐mouse: 5’‐AGGCTTCAGTGGTTTGGATG‐3’ (forward), 5’‐CACCAACAGCAC CATCGTTA‐3’ (reverse). Collagen III‐mouse: 5’‐CCCAACCCAGAGATCCC ATT‐3’ (forward).

### 2.9 Echocardiographic assessment

Echocardiography was performed in anesthetized mice using a Vevo 2100 Imaging System (VisualSonics, Inc) equipped with a 15‐MHz linear transducer. Enter B‐model mode to obtain the ventricular section at the level of the mitral papillary muscle. Systolic and diastolic ventricular wall thickness, ventricular diameter, and ventricular wall movement were monitored in M‐model mode. The percentage of fractional shortening (FS, %), ejection fraction (EF, %), and other parameters were obtained automatically.

### 2.10 Histological analysis

After being excised and washed with PBS, the hearts were fixed in 10% formalin and embedded in paraffin according to standard histological procedures. Subsequently, three tissue sections were selected from each animal for PSR, WGA, and TUNEL staining, respectively. Hearts were sectioned at 5‐μm‐thick. The sections were stained with PSR (Picrosirius Red) to evaluate collagen deposition and stained with phycoerythrin‐conjugated WGA (wheat germ agglutinin) (Invitrogen) to determine the cross‐sectional area of the myocytes. In addition, sections were stained with TUNEL for myocardial apoptosis detection. After staining with picrosirius, collagen deposition was measured by using Image J. The collagen volume fraction was determined as the percentage of picrosirius red positive‐stained area relative to total area. Similarly, after staining with phycoerythrin‐conjugated wheat germ agglutinin, the images of cardiomyocyte cross‐sectional areas were taken by fluorescence microscopy, and individual cell sizes were measured using a quantitative digital image analysis system (Image‐Pro Plus 6.0 software). More than 200 myocytes in the sections from eight different mouse samples were calculated in each group. For TUNEL count, cells with stained nuclei were determined by counting 100 cells in three randomly selected fields. The sections from eight different mouse samples were calculated in each group.

### 2.11 Statistical analysis

All quantitative data were expressed as means ± SD. Statistical comparisons between more than two groups were performed using two‐way analysis of variance (ANOVA); Data from two groups comparison were analyzed using unpaired Student's *t*‐test. All *p*‐values were two sides and *p*‐values <.05 were defined as statistical significance. All researchers were blinded throughout the experiments.

## 3 RESULTS

### 3.1 Honokiol attenuated intracellular oxidative stress induced by As_2_O_3_

**FIGURE 1:**
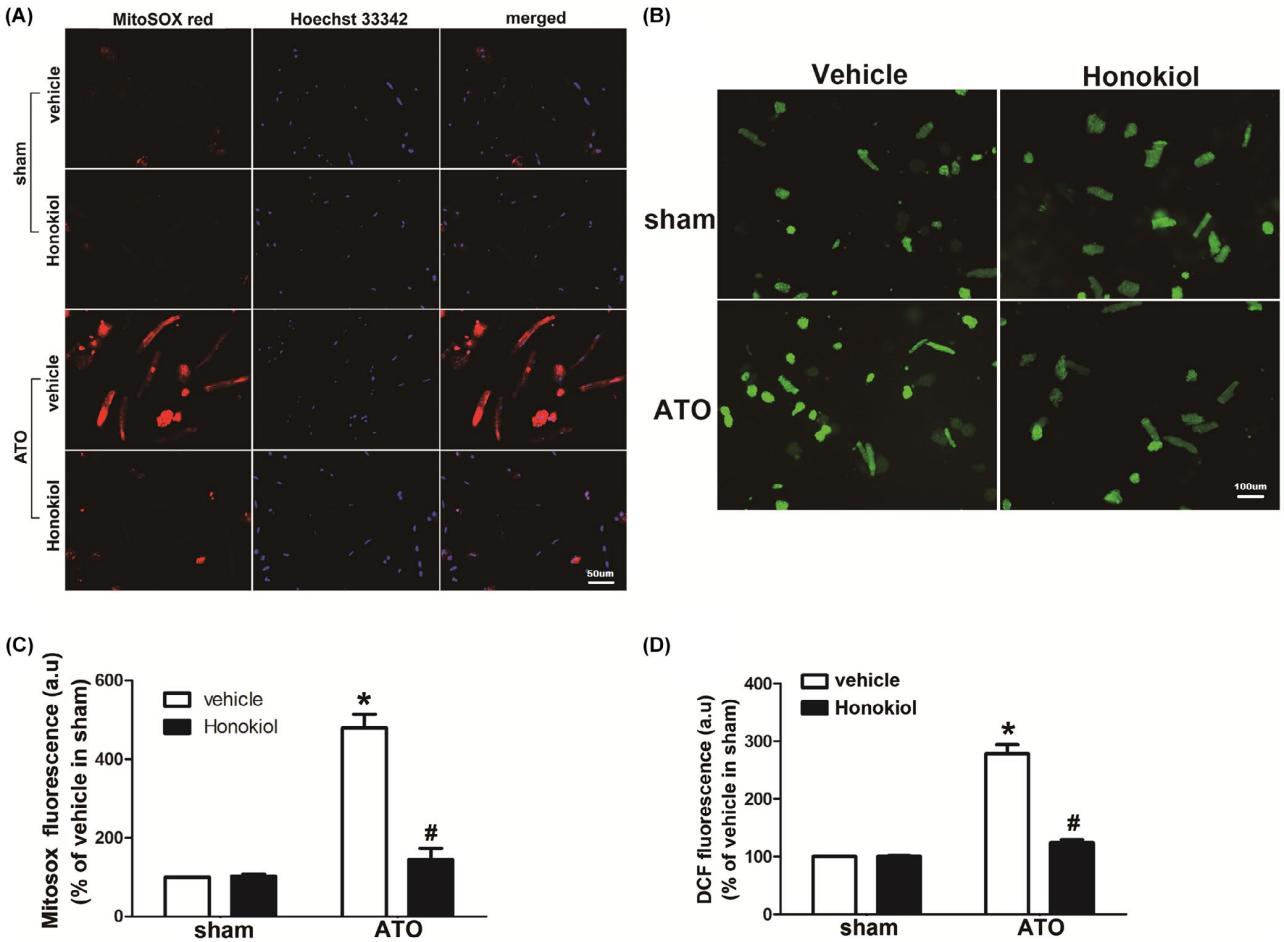
Effects of Honokiol on ATO‐induced reactive oxygen species (ROS) in primary cultured cardiomyocytes. Honokiol attenuated mitochondrial superoxide measured by using MitoSOX red. Cardiomyocytes were stained with Hoechst and MitoSOX red, 4 μM ATO caused an increased MitoSOX red fluorescence in primary cultured cardiomyocytes, which was attenuated by Honokiol pretreatment (10 μM), Representative image from five different cultures for the MitoSOX staining (A), The fluorescence intensity of MitoSOX Red was measured by Image J software (B); Similarly, Honokiol attenuated ATO‐induced oxidant generation. The intracellular ROS generation was determined by carboxy‐DCFDA. ATO induced an increase in DCF fluorescence, which was decreased by Honokiol retreatment, representative image from five different cultures for the DCFDA staining (C), the fluorescence density of DCF was acquired using fluorescence plate reader (D). The data are expressed as means ± SD from five independent cell cultures. *p < .05 versus vehicle in sham; ^#^ *p* < .05 versus vehicle in ATO‐treated cells

ROS generation has been found to be the major deleterious pathogenesis responsible for cardiomyocyte injury, it contributes to cellular damage and the apoptotic process. The main cardiotoxic effects of arsenic trioxide are mediated by superfluous boost in ROS concentration resulting in oxidative stress. ROS include radical species such as superoxide (O_2_•−), which cause oxidative damage to cellular macromolecules, including lipids, proteins, and polynucleotides. As illustrated in Figure 1A, MitoSOX red fluorescence, a mitochondrial‐targeted indicative of surplus superoxide production, was remarkably increased in ATO‐exposed cells compared to the control, indicating that mitochondrial superoxide generation was significantly boosted by ATO exposure. Nevertheless, Honokiol pretreatment markedly inhibited ATO‐induced mitochondria‐derived superoxide generation, as indicated by decreased MitoSOX red fluorescence to normal control level. In addition, we used DCF fluorescence to measure intracellular ROS generation by ATO exposure, Figure 1B showed that ATO treatment induced a significant increase in DCF fluorescence‐sensitive ROS in cardiomyocytes, but Honokiol pretreatment caused remarkable attenuation in DCF fluorescence. Honokiol alone had no effect on DCF fluorescence.

### 3.2 Honokiol decreased cardiomyocytes apoptosis induced by As_2_O_3_

**FIGURE 2:**
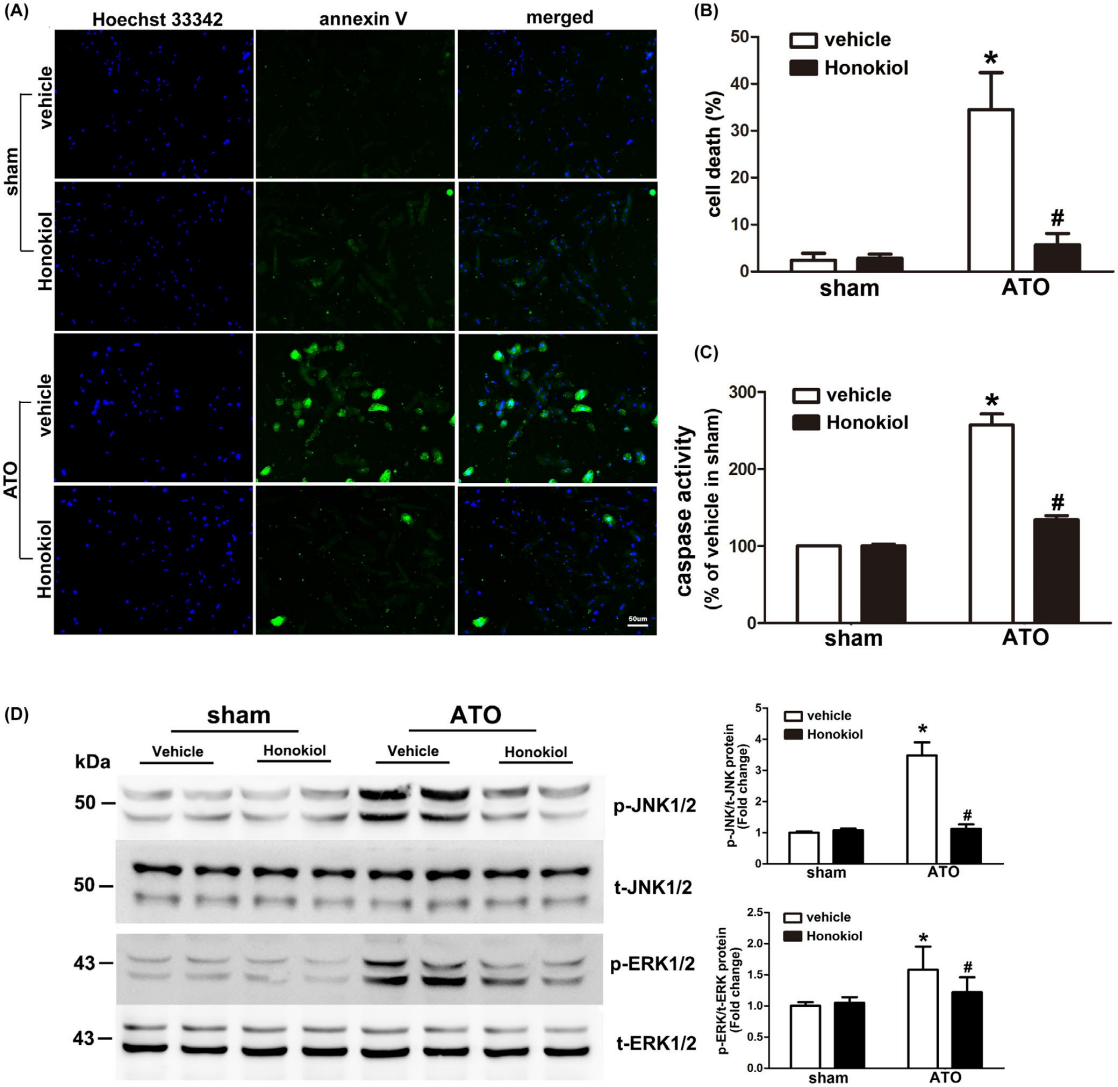
Effects of Honokiol on ATO‐induced apoptosis in primary cultured cardiomyocytes. Cardiomyocytes were pretreated with Honokiol for 6 h followed by sham or 4 μM ATO treatment, inhibition of ATO‐induced apoptosis by ATO was assessed through Hoechst 33342(blue)/annexin V (green) staining. Cell images were taken under a fluorescence and UV light microscope, blue spots represent cell nuclei and green spots represent apoptotic bodies (A); the rate of cell death was counted under microscope (B); activity of caspase‐3 enzyme was measured using a fluorometric assay (C). The expression levels of phosphorylated and total ERK1/2 and JNK1/2 were detected by western blot analysis. A representative western blot was shown, quantitative data of p‐JNK/T‐JNK (upper) and p‐ERK/T‐ERK (lower) ratio were shown at right side. Lysates from primary cultured cardiomyocytes was collected 24 h after treatment from each group. Honokiol pretreatment significantly attenuated ATO‐induced phosphorylation of JNK1/2 and slightly inhibited phosphorylation of ERK1/2, collectively (D). The data are expressed as means ± SD from five independent experiments. *p < .05 versus vehicle in sham; ^#^ *p* < .05 versus vehicle in ATO‐treated cells

To determine the protective effects of Honokiol against ATO‐induced cell death, primary cultured cardiomyocytes were pretreated with Honokiol for 6 h, and then treated with 4 μM ATO for 24 h. The induction of apoptosis was analyzed by Hoechst 33342/annexin V staining assays. As shown in Figure 2A, the number of annexin V positive cells increased following exposure to ATO, in contrast, in the presence of Honokiol, ATO‐induced apoptosis was significantly attenuated. Given that activation of the caspase cascade is crucial in the initiation of apoptosis, caspase‐3 activity was detected by fluorometric caspase‐3 assay kit. Our results (Figure 2C) show that caspase‐3 activity was significantly increased in primary cultured cardiomyocytes 24 h after exposure with 4 μM ATO, whereas the pretreatment of Honokiol significantly suppressed ATO‐induced caspase‐3 activation. In addition, we further investigated whether posttreatment of Honokiol could attenuate ATO‐induced cytotoxicity. The protective effects of Honokiol on cardiomyocytes after ATO‐treatment were measured by LDH leakage assay, caspase3 activity measurement, and annexin V staining. As shown in the Supplementary file, the co‐treatment group had a minor protective effect on ATO‐induced cardiac cytotoxicity. The posttreatment group had barely any effect on cardiotoxicity. Similarly, by using the caspase‐3 colorimetric assay kit to detect caspase‐3 activation in cardiomyocytes, we found that, compared with the control group and the Honokiol group, there was no significant improvement in the cardiotoxicity in the posttreatment group at each time course. Honokiol did not significantly improve the cardiotoxicity of ATO and was almost equal to caspase‐3 activation in the ATO group. Annexin V staining assays were consistent with the results of Caspase detection (Supplementary file). To investigate the signal transduction that participate in arsenic trioxide‐induced apoptosis of cardiomyocytes, we monitored activation status of MAPKs, including extracellular signal‐related kinases (ERKs), c‐jun NH_2_‐terminal kinases (JNKs), and p38 MAPKs, by their phosphorylation levels relative to the expression of total proteins in cardiomyocytes exposed to arsenic trioxide. Western blot analysis showed that arsenic trioxide treatment caused significant increase in the level of ERK1/2 and JNK1/2 phosphorylation, interestingly, prevention of cardiac apoptosis by Honokiol is accompanied by attenuated increase in p‐JNK1/2, as compared with As_2_O_3_ alone. Meanwhile, p‐ERK1/2 level increased by ATO exposure is inhibited in lesser extent (Figure 2D). However, there is lack of difference in p‐p38 expression in each group (data not shown).

### 3.3 Honokiol restored As_2_O_3_‐impaired cardiac function in mice

**FIGURE 3:**
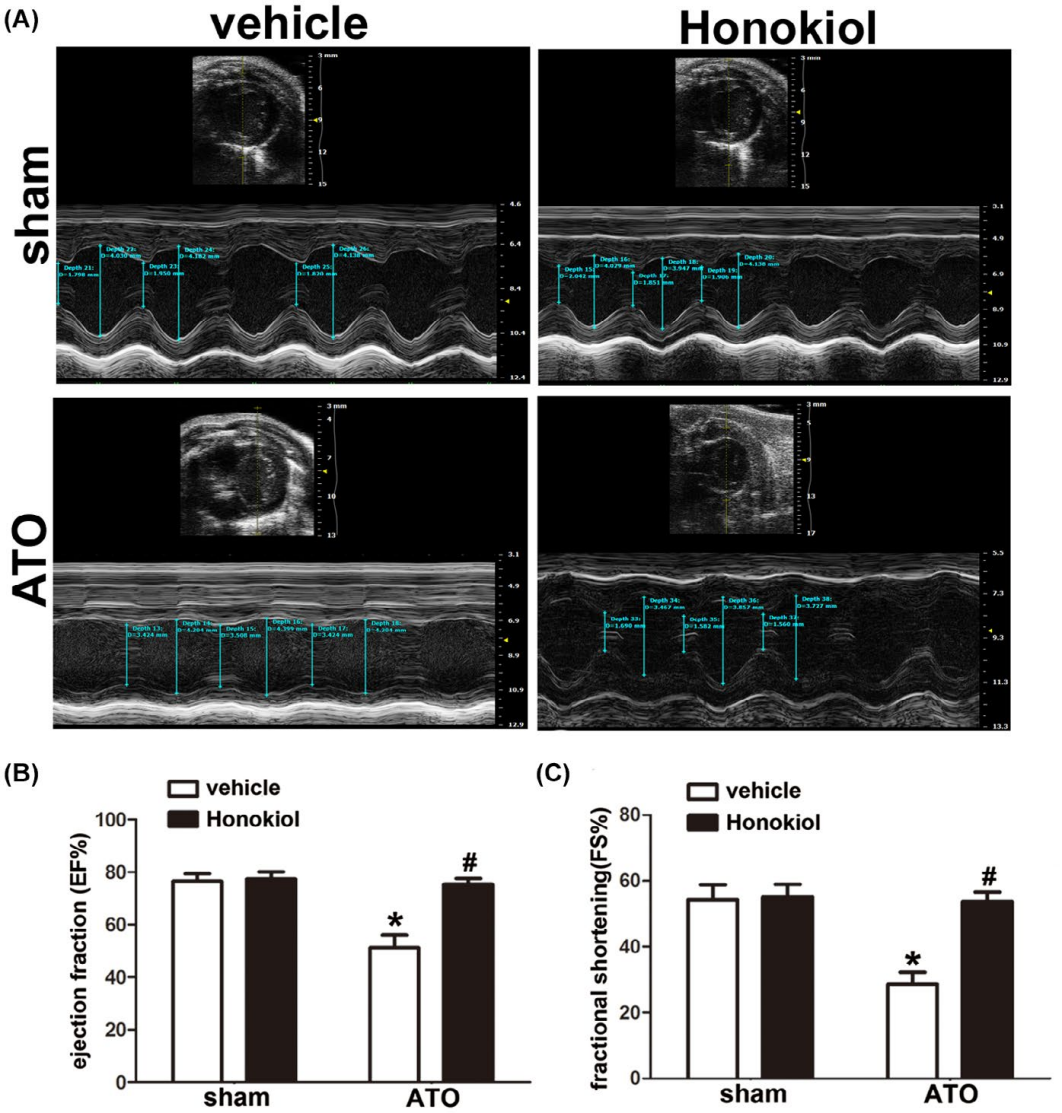
Effect of Honokiol on ATO‐induced cardiac dysfunction is determined by echocardiography. Twenty‐four hours after last treatment, all mice were anesthetized and subjected to echocardiography. Representative M‐mode echocardiographic photos of left ventricular chamber (A). Measurement of Ejection fraction (EF) in each group (B); Fraction shortening (FS) in each group (C). The data are expressed as means ± SD, *n* = 8, *p < .05 versus vehicle in sham; ^#^ *p* < .05 versus vehicle in ATO‐exposed mice

To evaluate the effect of Honokiol on the cardiac contractile function disturbed by ATO‐induced cardiotoxicity in vivo, mice were pretreated with vehicle or Honokiol at dose of 0.2 mg/kg and subjected to sham or ATO (4 mg/kg) administration, after 4 weeks of administration, M‐mode Echocardiography assessment was performed to evaluate the effect of ATO on cardiac function and protective role of Honokiol. The corresponding echocardiographic images (Figure 3A) showed that ATO exerts deleterious effect on cardiac function as indicated by significant declined ejection fraction (EF %) (Figure 3B) and fractional shortening (FS %) (Figure 3C), but Honokiol pretreatment markedly reversed the ATO‐induced falling‐off of the ejection fraction (EF %) and the fractional shortening (FS %).

### 3.4 Honokiol eliminated mitochondrial ROS generation and oxidative stress in ATO‐treated hearts

**FIGURE 4:**
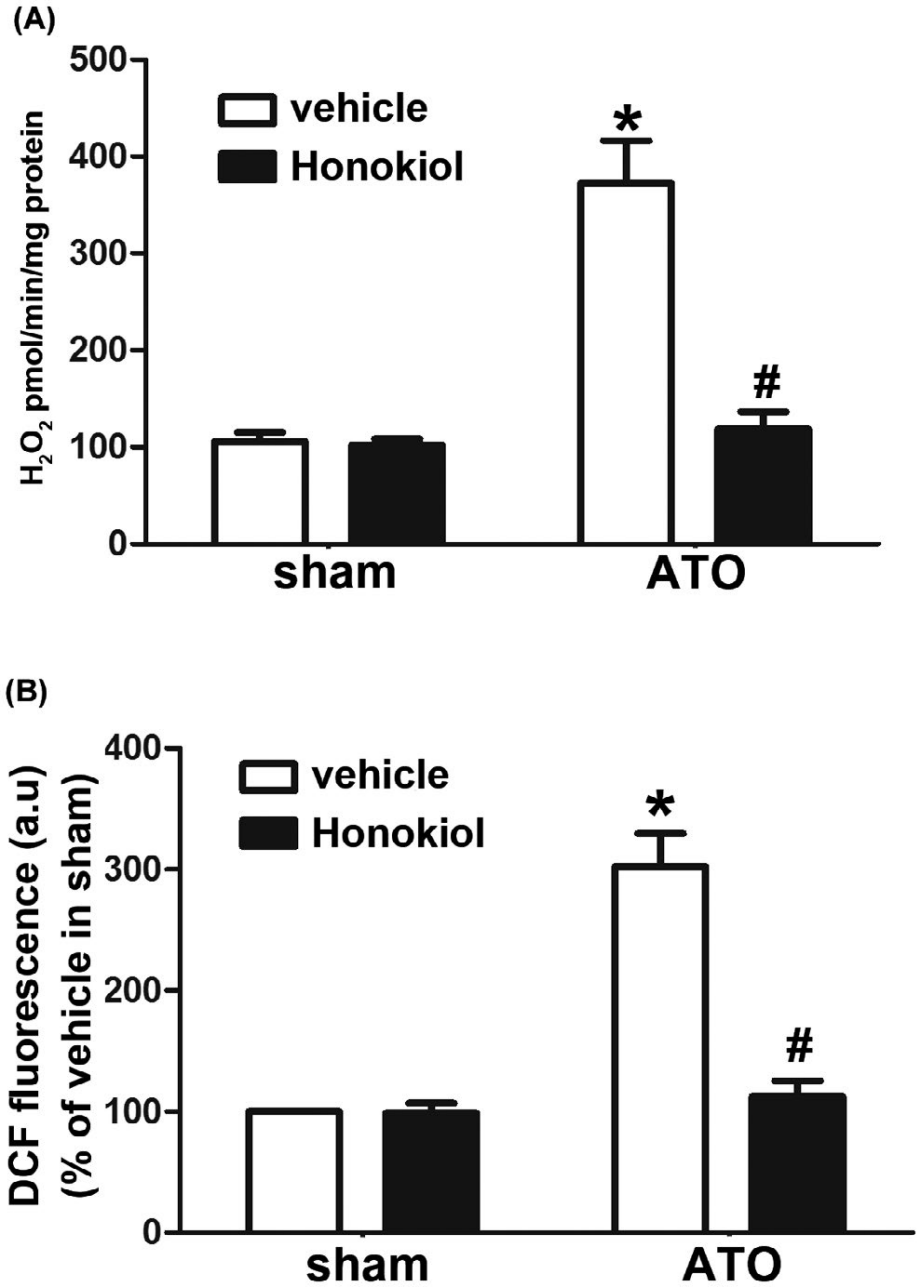
Effect of Honokiol on mitochondrial ROS generation and oxidative stress in ATO‐treated hearts. Honokiol attenuated ATO‐induced increase in ROS (H_2_O_2_) production in isolated heart mitochondria, as measured by Amplex Red (A), ROS generation in whole tissue lysates of hearts from each group was measured by DCFDA using fluorescence plate reader (B). The data are expressed as means ± SD, *n* = 6–8, *p < .05 versus vehicle in sham; ^#^ *p* < .05 versus vehicle in ATO‐exposed mice

We have shown that Honokiol can effectively reduce the ROS upregulation in cardiomyocytes exposed to arsenic trioxide. Similarly, we investigated ROS levels in heart tissues of animal experiment. We first assessed the effect of ATO and (or) HKL on production of ROS in isolated mouse heart mitochondria using Amplex Red. The results showed a substantial increase in ROS (H_2_O_2_) production in isolated heart mitochondria compared to sham and HKL group. In contrast, pretreatment of HKL significantly decreased mitochondrial ROS (H_2_O_2_) generation (Figure 4A). And then, the total oxidative stress levels in heart tissue were also measured by DCF fluorescence, as tested in primary cultured cardiomyocytes. As illustrated in Figure 4B, ATO treatment induced a significant increase in DCF fluorescence‐sensitive ROS in heart tissue lysates, whereas HKL pretreatment caused remarkable attenuation in DCF fluorescence. These data indicated that HKL effectively eliminates the mitochondrial ROS production as well as oxidative stress in ATO‐treated mouse hearts.

### 3.5 Honokiol inhibited the myocardial apoptosis in mice exposed to As_2_O_3_

**FIGURE 5:**
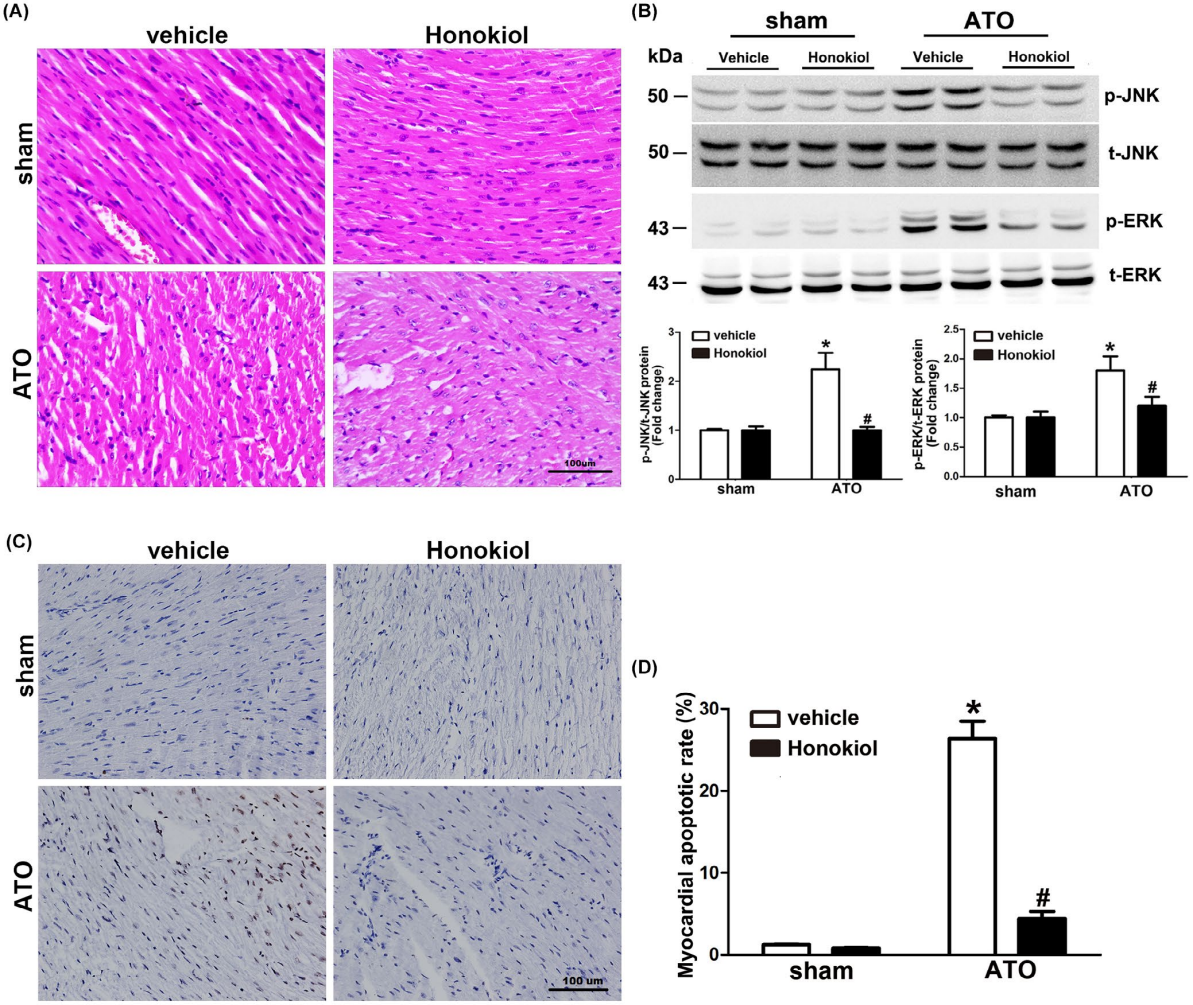
Effect of Honokiol on myocardial apoptosis in mice exposed to ATO. Myocardial tissue sections of left ventricular specimens stained with HE (A), the characteristics of ATO cardiotoxicity were found in left ventricular sections, while HKL pretreatment abrogated the pathological changes made by ATO. Heart lysates were analyzed by western blotting for the phosphorylated and total ERK1/2 JNK1/2, Myocardial ERK1/2, and JNK1/2 phosphorylation level were markedly increased by 4 weeks of ATO exposure, which was inhibited by HKL pretreatment (B). Myocardial tissue sections of left ventricular specimens stained with TUNEL (C), heart sections from ATO group exhibited higher percentage of apoptotic cells. Pretreatment with HKL significantly decreased the percentage of apoptotic myocardial cells (Magnification = 200×). Apoptosis index was counted by two independent pathologists (D). The data are expressed as means ± SD, *n* = 6–8, *p < .05 versus vehicle in sham; ^#^ *p* < .05 versus vehicle in ATO‐exposed mice

We have demonstrated that Honokiol could effectively reduce apoptosis induced by arsenic trioxide in primary cultured cardiomyocytes. To further confirm the cardio‐protective effect of HKL, the myocardial tissues were stained with HE and TUNEL. HE staining (Figure 5A) displayed that myocardial tissue structure of both sham group and HKL group were healthy, with orderly arrangement of myocardial fibers, intact structure of cell membrane, and uniform size of nuclei. However, myocardial fibers arrangement in ATO group was disordered, different nuclear sizes and karyopyknosis, myocardial edema and cell membranes collapse could be observed. In contrast, treatment with HKL apparently protected heart tissues against the ATO‐induced myocardial pathological changes. To evaluate the effect of ATO or/and HKL on cardiomyocyte apoptosis in myocardial tissues, TUNEL staining was carried out (Figure 5B,C). Compared with myocardial tissues in sham and HKL group, myocardial tissues in ATO group exhibited significant higher percentage of apoptotic cells. Pretreatment with HKL almost abolished myocardial apoptosis, indicating that HKL could provide cardio‐protective effects against ATO‐induced toxicity through repressing myocardial apoptosis. Corresponding to in vitro experiments, HKL also attenuated increase in the level of ERK1/2 and JNK1/2 phosphorylation induced by long‐term ATO administration in myocardial tissues (Figure 5D).

### 3.6 Honokiol ameliorated As_2_O_3_‐induced cardiac hypertrophy

**FIGURE 6:**
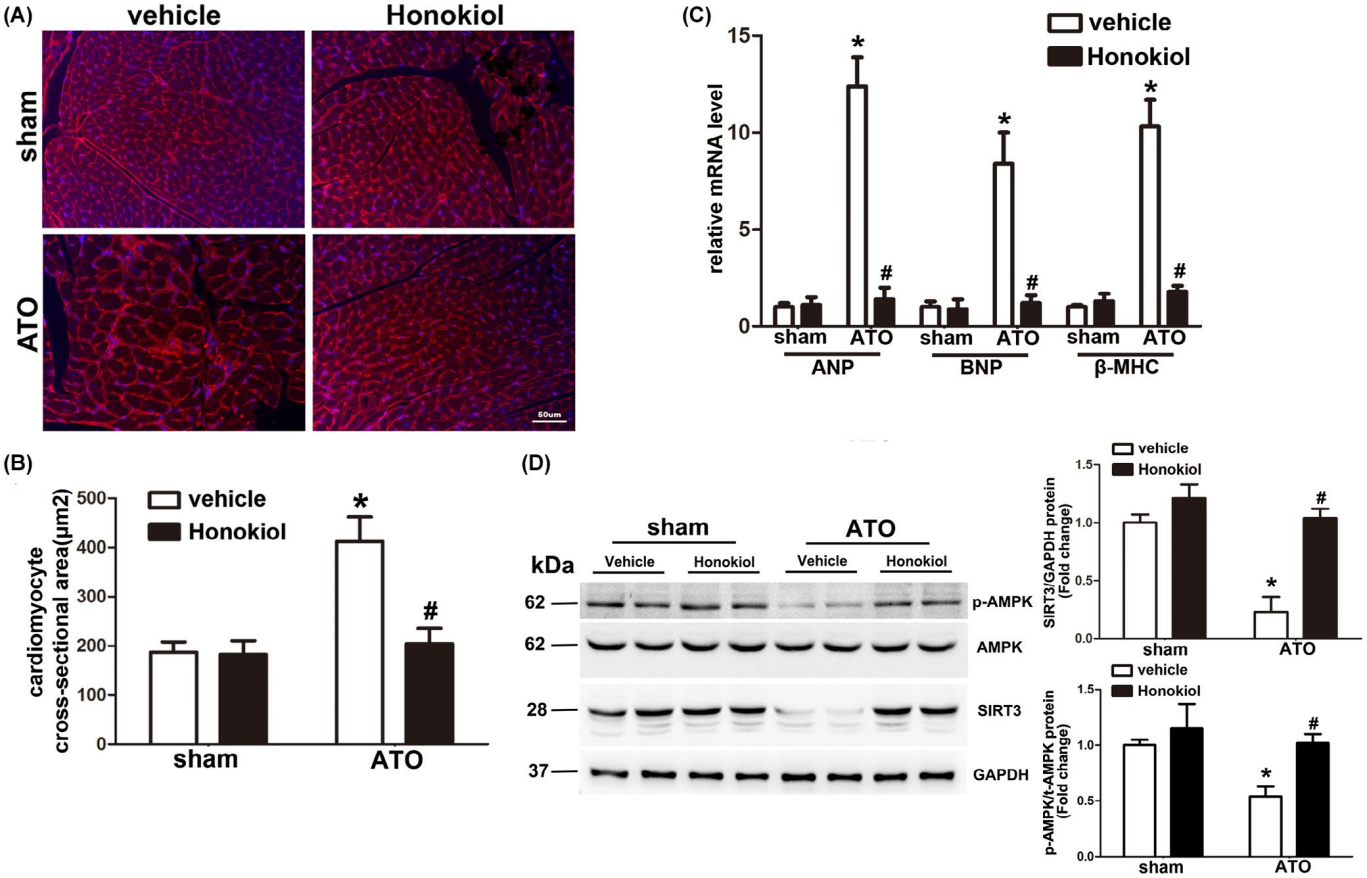
Honokiol pretreatment attenuated cardiac hypertrophy induced by arsenic trioxide exposure. Representative images of heart sections stained with WGA (red) and Hoechst 33342(blue) showed that cell size of cardiomyocytes increased by 4 weeks of ATO exposure, which was obviously attenuated by Honokiol pretreatment (A). Statistical results for quantification of the cardiomyocyte size in cross‐sectional area (B). Quantification results for real‐time PCR analysis of hypertrophic marker genes including ANP, BNP, and β‐MHC from hearts of mice in the indicated groups (C). Heart lysates were analyzed by western blotting for the indicated antibodies. Myocardial AMPK phosphorylation level and the expressions of SIRT3 were markedly downregulated by 4 weeks of ATO exposure, which was reversed by Honokiol pretreatment (D). The data are expressed as means ± SD, *n* = 6–8, *p < .05 versus vehicle in sham; ^#^ *p* < .05 versus vehicle in ATO‐exposed mice

To determine whether Honokiol pretreatment could antagonize arsenic trioxide‐induced cardiac hypertrophy and remodeling, sections of heart in each group were stained with wheat germ agglutinin (WGA) (Figure 6A), and then image digital analysis system revealed that cardiomyocyte cross‐sectional area increased by more than four times in mice exposed to ATO compared with sham control, and this effect was obviously reduced by Honokiol pretreatment (Figure 6B). In addition, the hallmarks of cardiac hypertrophy, atrial natriuretic peptide (ANP), B‐type natriuretic peptide (BNP), and β‐myosin heavy chain (β‐MHC) mRNA expression were also in line with that of histological analyses: Honokiol pretreatment markedly reversed the upregulation of ANP, BNP, and β‐MHC levels, as indicated in Figure 6C. Collectively, these data indicated that Honokiol pretreatment improved ATO‐induced cardiac hypertrophic remodeling. Given that Honokiol is the pharmacological activator of Sirt3,^19^ and AMPK‐SIRT3 axis has pivotal role in pathophysiological process of cardiac hypertrophy, we analyzed the expression profile of AMPK and SIRT3 in heart lysates of each group. Western blot data (Figure 6D) showed that myocardial AMPK phosphorylation level and the expressions of SIRT3 were markedly downregulated by ATO exposure, however, Honokiol pretreatment significantly reversed these decreases, indicating that Honokiol exerted‐protection of cardiac hypertrophy during ATO‐induced cardiotoxicity is mediated through activating the Sirt3/AMPK signaling pathway.

### 3.7 Honokiol attenuated As_2_O_3_‐induced cardiac fibrosis

**FIGURE 7:**
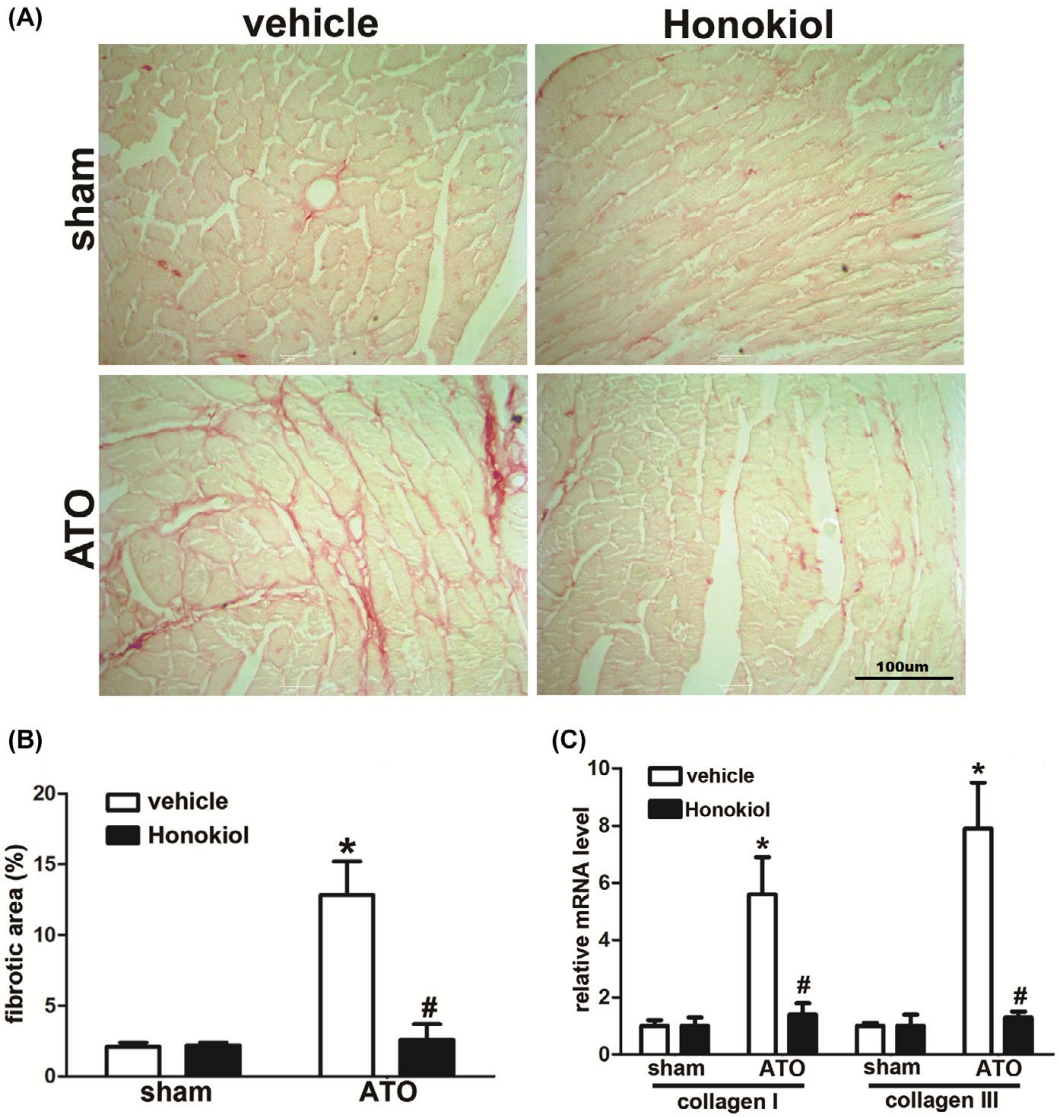
Arsenic trioxide exposure causes cardiac hypertrophy in mice that is reduced upon Honokiol pretreatment. Representative images of histological analysis of cardiac interstitial fibrosis, picrosirius red staining shows that the cardiac fibrosis increases in arsenic‐treated mice heart tissue compared to control after 4 weeks of treatment. The fibrosis level is reduced in case of Honokiol pretreatment (A). Statistical results for the fibrotic area (*n* > 40 fields per group) (B), quantification results for mRNA levels of the fibrotic marker genes (collagen I, collagen III) in the indicated groups (C). The data are expressed as means ± SD, *n* = 6–8, *p < .05 versus vehicle in sham; ^#^ *p* < .05 versus vehicle in ATO‐exposed mice

Cardiac fibrosis is a common pathological feature accompanied with cardiac hypertrophy, which results from the disruption of normal cardiac structures and functions due to the interstitial fibroblasts accumulation and excessive deposition of extracellular matrix (ECM) proteins in the myocardium. It is been revealed that ATO‐induced cardiac fibrosis is associated with the development of long QT syndrome, indicating that cardiac fibrosis also plays a critical role in ATO‐induced cardiotoxicity. To verify if Honokiol pretreatment could attenuate As_2_O_3_‐induced cardiac fibrosis, paraffin‐embedded slides were stained with picrosirius red (PSR). As illustrated by Figure 7A,B, conspicuous interstitial fibrosis was detected in ATO‐treated mice, but the development of cardiac fibrosis was obviously prevented by Honokiol pretreatment. Consistently, subsequent analysis of mRNA levels of marker genes collagen I, collagen III, which are responsible for cardiac fibrosis, showed the similar results (Figure 7C).

## 4 DISCUSSION

In the present study, we sought to investigate the protective effect of Honokiol on arsenic trioxide‐induced cardiotoxicity both in vitro and in vivo. In primary culture of cardiomyocytes treated with ATO in the presence or absence of HKL, we found that HKL pretreatment apparently helped in protecting cardiomyocytes from ATO‐induced apoptosis. To further elucidate the underlying mechanism, we measured the mitochondrial ROS level following exposure to ATO after treatment with HKL. As indicated by MitoSOX Red and Amplex Red, HKL pretreatment significantly attenuated ATO‐induced elevation of mitochondrial ROS level, which was associated with its anti‐apoptotic activity. Consistent with the in vitro study, we also demonstrated that HKL treatment could ameliorate arsenic trioxide‐induced cardiac hypertrophy and cardiac fibroblast proliferation in mice. These data provided the mechanistic link between pathological alterations induced by arsenic trioxide and the pharmacological mechanisms of Honokiol.

Due to the high energy consumption, cardiomyocytes have high mitochondrial densities which account for up to 40% of total cellular volume, and are thus particularly susceptible to mitochondrion‐toxic agents.^9^ It is been verified that the cardiotoxicity of arsenic trioxide is mediated mainly through mitochondrial dysfunction by interfering with electron transport system, which result in disruption of cellular redox homeostasis followed by rapid boost in ROS generation.^15^ Excessive ROS generation induced by arsenic trioxide has a central role in initiation and progression of cardiac cytopathic via multitude of downstream effects.^21^ Hence, agents with the ability to scavenge or reduce ROS concentrations may be therapeutically useful for patients who require arsenic trioxide therapy for APL or other cancers. Honokiol molecule possess two phenolic groups which can scavenge free radicals and thus exhibit antioxidant property.^22^ It has been reported that Honokiol protected rat hearts against myocardial ischemia reperfusion injury by attenuation of oxidative stress and concomitant NF‐κB activation, reducing the ROS‐induced activation of apoptotic pathways.^17^ In the current study, consistent with the mechanism whereby ATO exposure cause ROS‐mediated cardiotoxicity, we found that ROS level was remarkably increased after ATO treatment as measured by DCF fluorescence, Nevertheless, our result also demonstrated that pretreatment with Honokiol could prevent the ROS overproduction in ATO‐treated cardiomyocytes. Superoxide (O_2_•−) is the primary ROS formed inside mitochondria,^23^ therefore we examined if mitochondrial O_2_•− could be detoxified by Honokiol pretreatment. As measured by MitoSOX and Amplex Red, arsenic trioxide induced an increase in the red fluorescence, which was reduced by Honokiol to almost that of controls, suggesting Honokiol predominantly functions in neutralizing excessive mitochondrial O_2_•−. Increased ROS generation mediated by arsenic trioxide disrupts mitochondrial membrane and releases the cytochrome c, triggering caspase‐3 activation as the major cause of intrinsic apoptotic pathways.^24^, ^25^ In this regard, a recent study showed that Honokiol protects the heart against Dox‐induced cardiac pathological development by improving mitochondrial respiration and reducing cardiac apoptosis.^19^ Given that cardiac toxicity caused by ATO shares a similarity with that of doxorubicin via intrinsic and extrinsic pathways of apoptosis, it is reasonable to speculate that Honokiol could ameliorate the cardio‐toxic effects posed by ATO in a similar manner. In line with previous studies, the current study also demonstrated that ATO treatment leaded to cardiomyocyte apoptosis, in contrast, Honokiol pretreatment strongly reversed ATO‐induced apoptosis that were characterized by reduced annexin V‐positive cells and inhibited caspase‐3 activity. Mitogen‐activated protein kinases (MAPKs), which consist of ERK1/2, JNK, and p38 MAPK, are vital mediators of apoptosis under various pathophysiological conditions including oxidative stress.^26^ The role of these kinases in the pathogenesis of ATO‐induced cardiotoxicity has been reported earlier.^27^, ^28^ In current study, we observed an obvious increase in phosphorylation of JNKs in ATO intoxicated cardiomyocytes, which was abrogated by Honokiol pretreatment. Taken together, these findings imply that Honokiol suppress arsenic trioxide‐induced cardiomyocyte apoptosis by inhibiting ROS‐stimulated downstream JNK signaling.

As a major risk factor for the development of heart failure, myocardial hypertrophy is an adaptive pathophysiological progression in response to a wide variety of intrinsic and extrinsic stimuli.^29^ During the transition from initially adaptive hypertrophy to decompensated heart failure, dilation of individual cardiomyocytes is concurrent with activation of canonical programmed cell death or apoptosis, resulting in cumulative dropout of cardiomyocytes and subsequent replacement of fibrosis within the myocardial matrix.^30^, ^31^ Therefore, strategies that prevent programmed loss of cardiomyocytes held promise in overcoming the maladaptive hypertrophy and heart failure. At molecular level, several apoptotic signaling pathways in cardiomyocyte undergoing pathological hypertrophy have been proposed, such as: activation of NF‐κB and its downstream signal pathway via ROS generation by TNF‐α or angiotensin‐Ⅱ^32^, ^33^; upregulation of apoptosis‐inducing factor and proapoptotic effector proteins (Bax, Bad)^34^; dysfunction in cytochrome P450 and its associated arachidonic acid metabolism^35^; involvement of MicroRNAs and its interaction with target posttranscriptional gene.^36^ In the case of arsenic‐induced cardiovascular diseases, the effect of arsenic exposure on cardiac hypertrophy has been previously reported.^37^ Chronic arsenic exposure‐induced cardiac hypertrophy is not only correlated with long‐term hypertension secondary to cardiovascular smooth muscle endothelial dysfunction, but also results from the direct cytotoxicity on cardiomyocytes of heart.^30^ In the present study, ATO‐treated C57Bl/6 mouse have exhibited the induction of cardiac hypertrophy, with obvious morphological features of myocardial hypertrophy and significant induction of hypertrophic markers ANP, BNP, and β‐MHC. A recent study has shown that arsenic trioxide‐induced cardiac hypertrophy both in vivo in mice and in vitro in rat H9c2 cardiomyocytes. Meanwhile, AMP‐activated protein kinase (AMPK), a serine‐threonine kinase that plays an important protective role in limiting apoptotic activity, was inhibited and thereby activating calcineurin/NFAT pathway.^37^ In a similar manner, our data indicated that the pathogenesis involved in arsenic‐induced cardiac hypertrophy is in obvious association with the induction of cardiomyocyte apoptosis via elevation of oxidative stress. It is been demonstrated that activation of AMPK‐PGC‐1α‐SIRT3 signaling pathway could preserve myocardial function by reducing mitochondrial oxidative stress and enhancing mitochondrial biogenesis in the context of cardiac hypertrophy.^38^ Therefore, utilization of cardio‐protective agent that target AMPK‐SIRT3 axis is supposed to be a novel therapeutic strategy to ameliorate ATO‐induced cardiac hypertrophy. Honokiol has been identified as an activator of SIRT3, which directly enter into mitochondria and bind to SIRT3 to enhance its enzymatic activity, thereby protecting the heart from pressure overload and doxorubicin‐induced cardiac hypertrophy.^19^, ^39^ In current study, we found that Honokiol could effectively attenuate ATO‐induced cardiac hypertrophy and dysfunction. During this pathological and pharmacological process, downregulation of myocardial AMPK phosphorylation level and expressions of SIRT3 by ATO exposure is reversed by Honokiol pretreatment. Therefore, SIRT3 may be a direct target of Honokiol in ATO‐induced cardiac toxicity. However, the entire mechanism by which Honokiol provides protection is yet to be fully understood. Thus our next research will be focusing on the HKL‐SIRT3‐related signaling pathway.

Cardiac fibrosis plays a critical pathophysiological role in the initiation and development of heart disease, which is characterized by interstitial fibroblasts accumulation and excessive deposition of extracellular matrix (ECM) proteins in the myocardium, leading to distorted cardiac architecture and impaired mechano‐electric coupling of cardiomyocytes.^40^ A previous study revealed that ATO‐induced interstitial myocardial fibrosis resulted from aberrant paracrine of various pro‐inflammatory cytokines and pro‐fibrotic factors by cardiac fibroblasts and culminated in the development of long QT syndrome.^41^ Therefore, exploring novel agents that can prevent the proliferation and differentiation of cardiac fibroblasts into myofibroblasts, as well as increased secretion of extracellular matrix proteins from cardiac fibroblasts, is helping in prevention and cure of cardiac fibrosis. This is promising, in light of the previous study demonstrating that Honokiol was capable of blocking Angiotensin‐Ⅱ‐induced differentiation of cardiac fibroblasts into myofibroblasts in SIRT3‐dependent manner.^39^ In our study, the extent of cardiac fibrosis and collagen secretion induced by ATO was strikingly prevented following Honokiol pretreatment, although the underlying mechanism has yet to be fully elucidated. Our further work will investigate the direct relationship between Honokiol and cardiac fibroblasts after ATO exposure, in parallel, it should also be explored that whether Honokiol had beneficial effect on transformation of vascular endothelial cells into myofibroblasts (endothelial‐to‐mesenchymal transition (EndMT)) triggered by ATO exposure.^42^

Although arsenic trioxide has been verified to cause cardiovascular toxicity, it is still widely used because they are highly effective in treatment of acute promyelocytic leukemia.^1^ Thus, it is imperative to search for novel therapeutic interventions to prevent and/or treat arsenic trioxide‐induced cardiotoxicity without compromising its anti‐APL effects based on the cellular and molecular basis of cardiac toxicology. The current study for the first time revealed that Honokiol pretreatment exerts protective effect on cardiomyocyte and cardiac tissue against ATO‐induced cardiotoxicity, which is attributed to inhibiting cardiomyocyte apoptosis via reduced oxidative stress. Intriguingly, Honokiol has shown to be an effective lignan in inducing cell death of human APL cells without inducing myelosuppression and immunodeficiency.^43^, ^44^ Therefore, it may open a possibility that combined application of arsenic trioxide and Honokiol can be a promising avenue in synergistic killing of acute promyelocytic leukemia cells.

## AUTHOR CONTRIBUTIONS

A.‐L.H. and F.Y. designed and conducted the experiments and wrote the paper. L. Zhou, L. Zhang, T.C. and D.P. conducted the experiments. X.J., L.M., X.X. performed the data analysis. F.Y., P.C. designed the experiments and supervised the research. All authors discussed the results and commented on the manuscript.

## DISCLOSURE

The authors declare that they have no conflict of interest.

## ETHICAL APPROVAL AND CONSENT TO PARTICIPATE

Ethical approval for the study was obtained by the Ethics Committee of State Key Laboratory of Biotherapy, Sichuan University.

## Supporting information

Figure S1–S7

## Data Availability

The data that support the findings of this study are available from the corresponding author upon reasonable request.
