# Supplementary material for: Honokiol attenuate the arsenic trioxide‐induced cardiotoxicity by reducing the myocardial apoptosis
Source: Pharmacol Res Perspect. 2022 Feb 16;10(2):e00914. doi: 10.1002/prp2.914 (PMC8848632; doi:10.1002/prp2.914)

Supplementary figure 1

The cultured cardiomyocytes were administrated with HKL(10μM) at 0, 2, 6, 12 hours after treated with ATO(4 μM). The protective effects of Honokiol on cardiomyocytes after ATO-treatment were measured by LDH leakage assay(A), caspase3 activity measurement(B), and annexin V staining(C) at each time-point.These results clearly showed that the HKL had a minor protective effect on ATO-induced cytotoxicity in the co-treated group (0 hours).However, the post-treatment of HKL (2, 6, 12 hours) had barely any protective effect on cardiotoxicity.


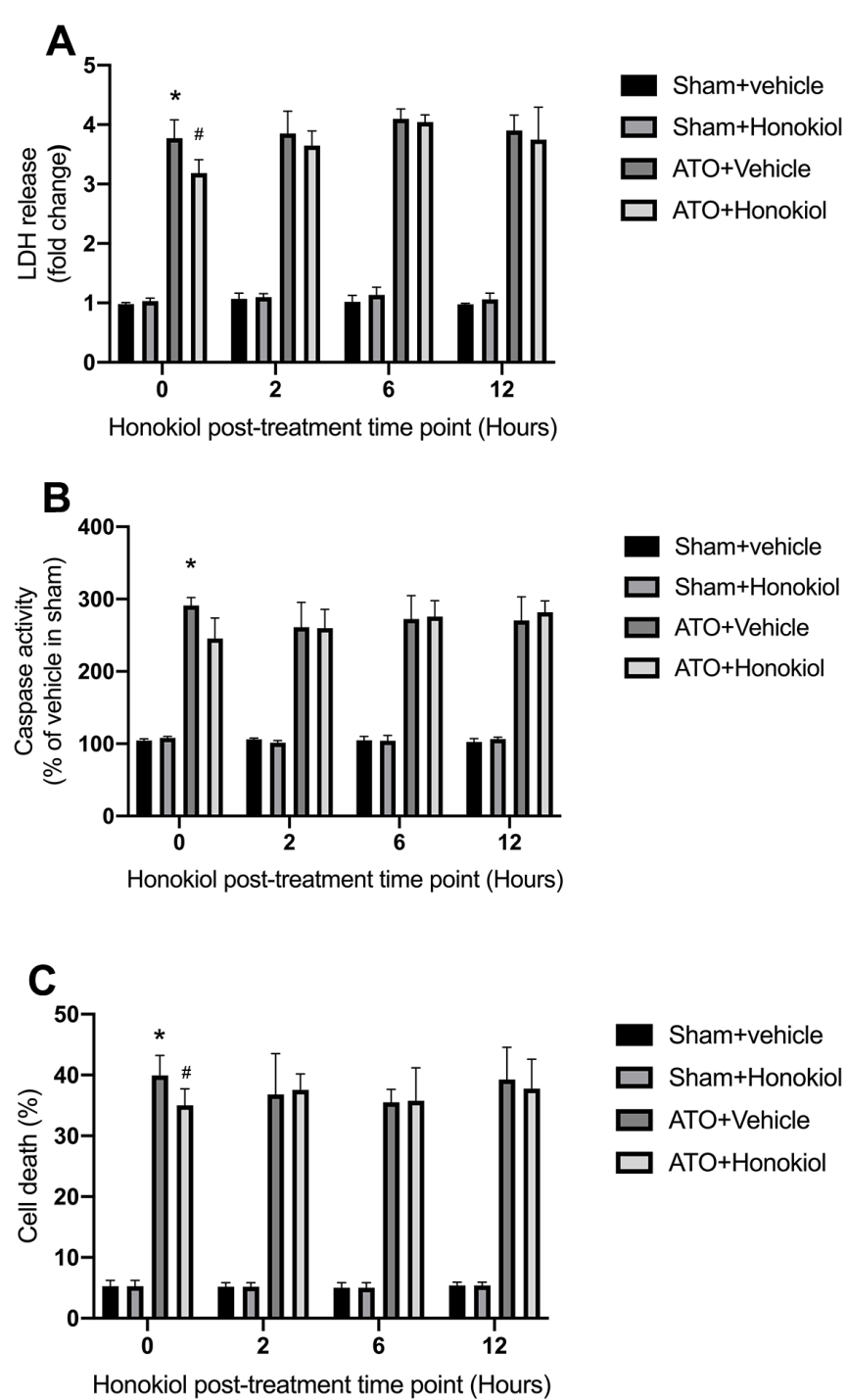


Supplementary figure 2

The expression levels of phosphorylated(A, C) and total JNK1/2(B, D) for in vitro study. A and B were used as representative graphs.


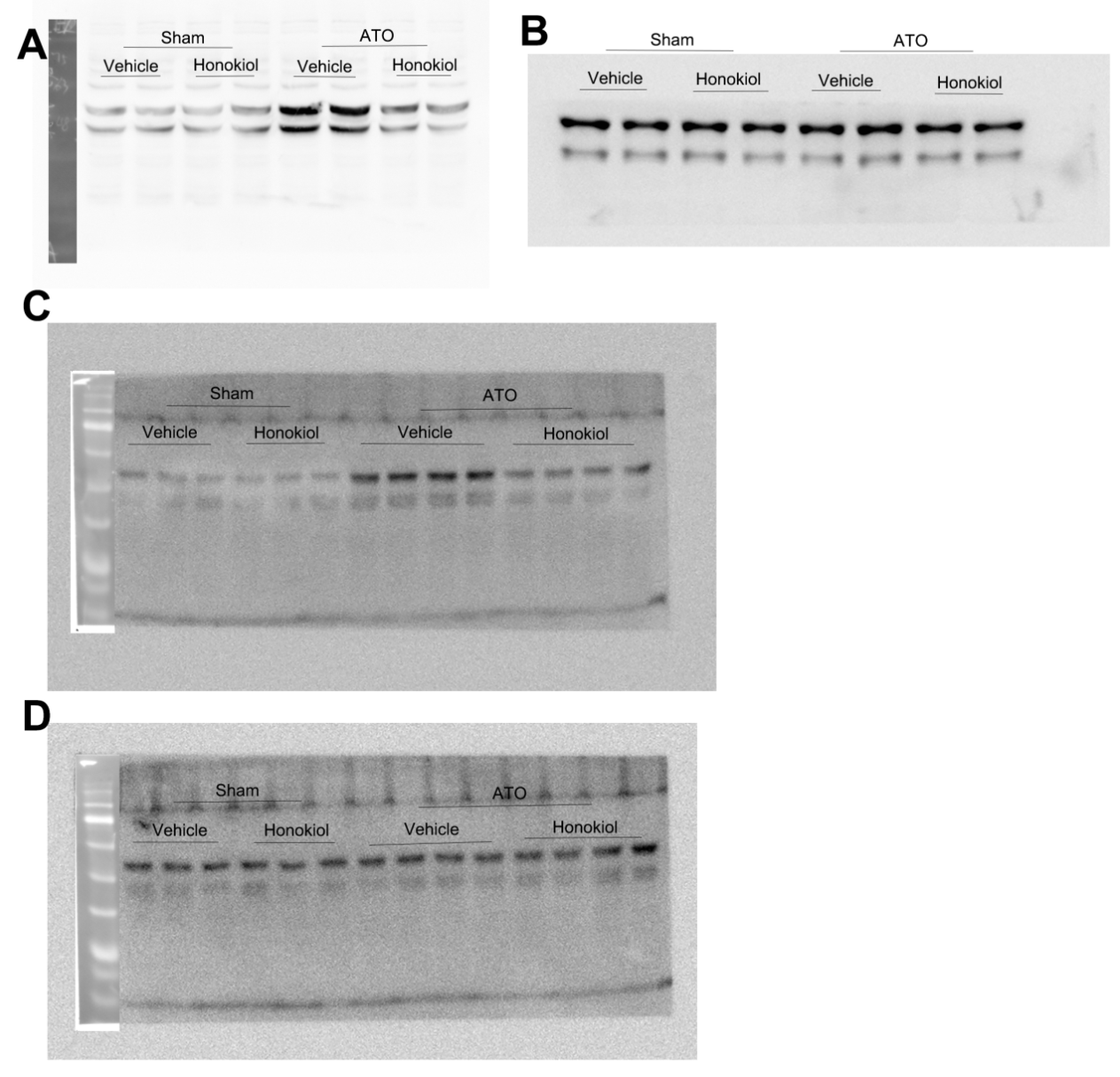


Supplementary figure 3

The expression levels of phosphorylated(A, C) and total JNK1/2(B, D) for in vivo study. A and B were used as representative graphs. A and B were used as representative graphs.


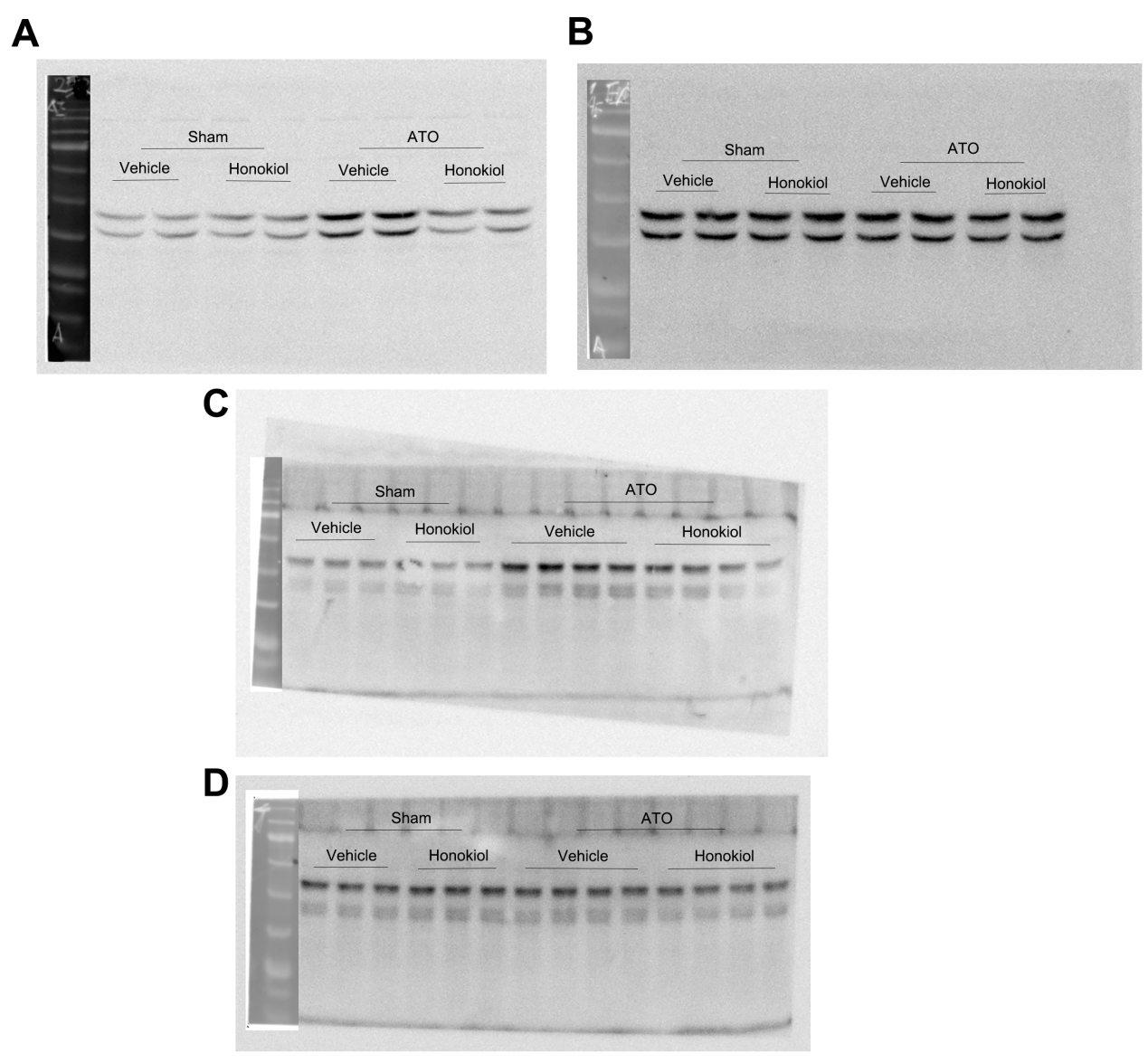


Supplementary figure 4

The expression levels of phosphorylated(A, B, C) and total ERK1/2(D, E, F) for in vitro study. B and D were used as representative graphs.


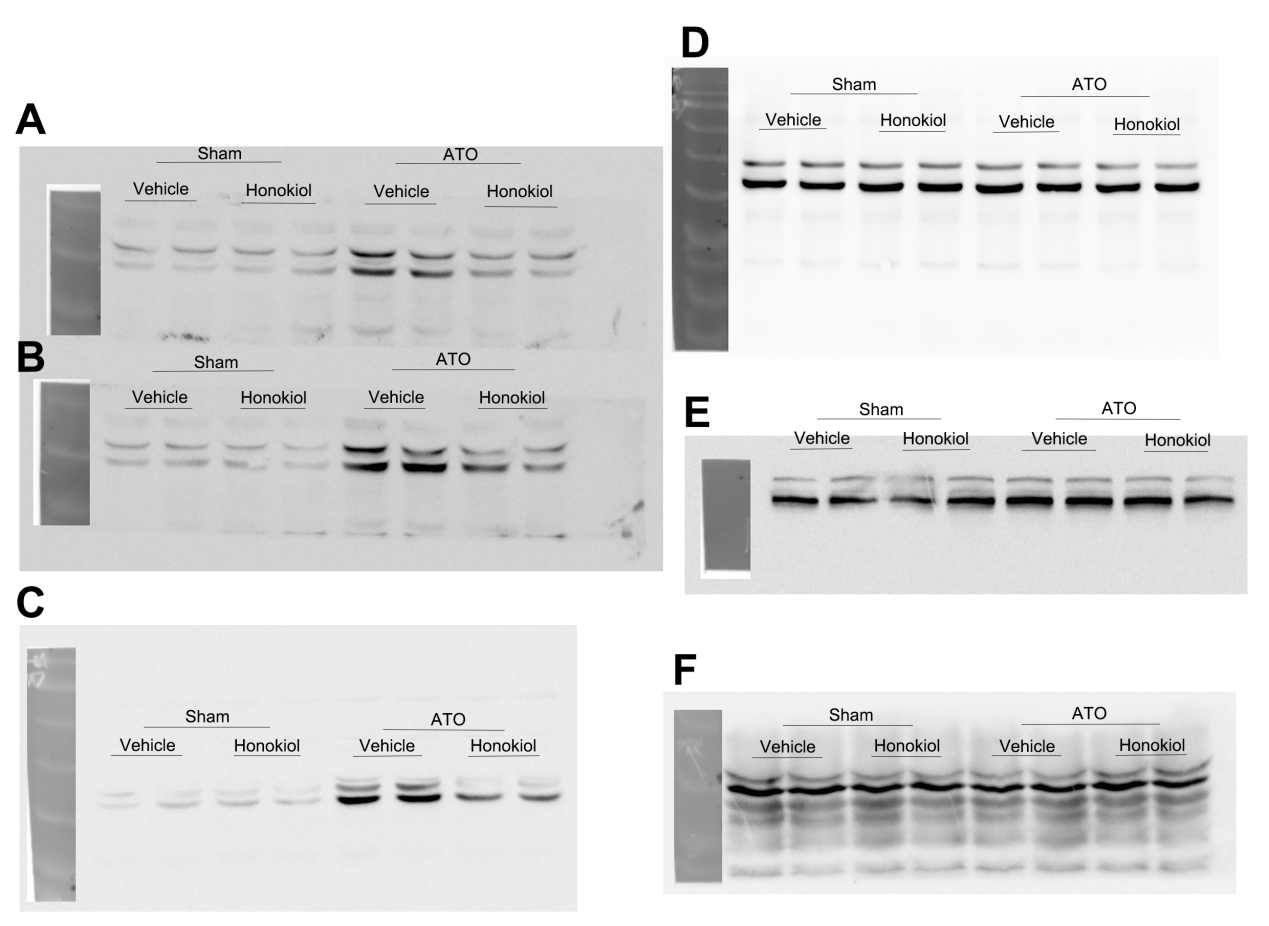


Supplementary figure 5

The expression levels of phosphorylated(A, B, C) and total ERK1/2(D, E, F) for in vitro study. B and D were used as representative graphs.


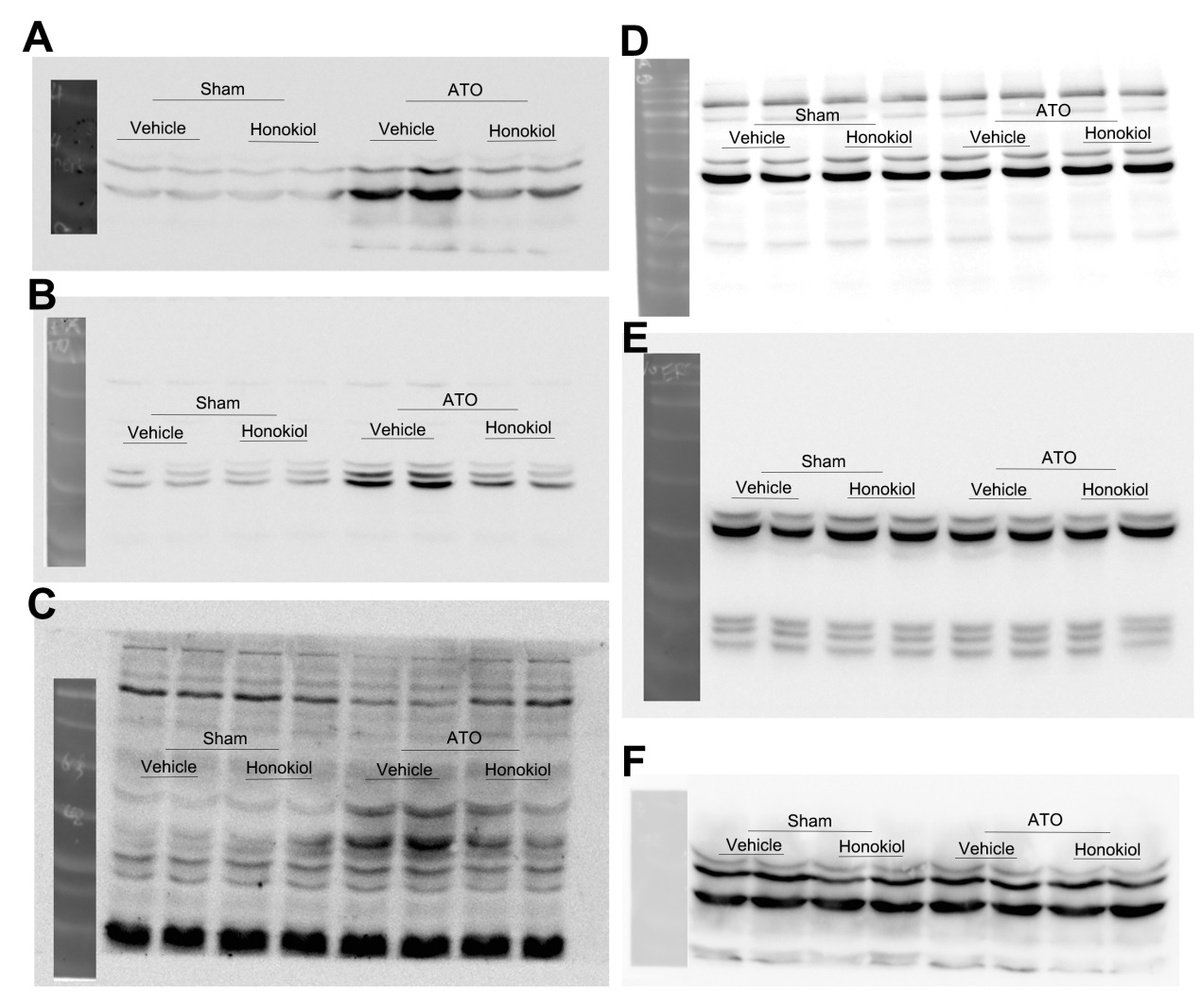


The expression levels of phosphorylated(A, B, C) and total ERK1/2(D, E, F) for in vivo study. B and D were used as representative graphs.

Supplementary figure 6

The expression levels of phosphorylated AMPK (A, B, C) and total AMPK (D, E, F). C and D were used as representative graphs.


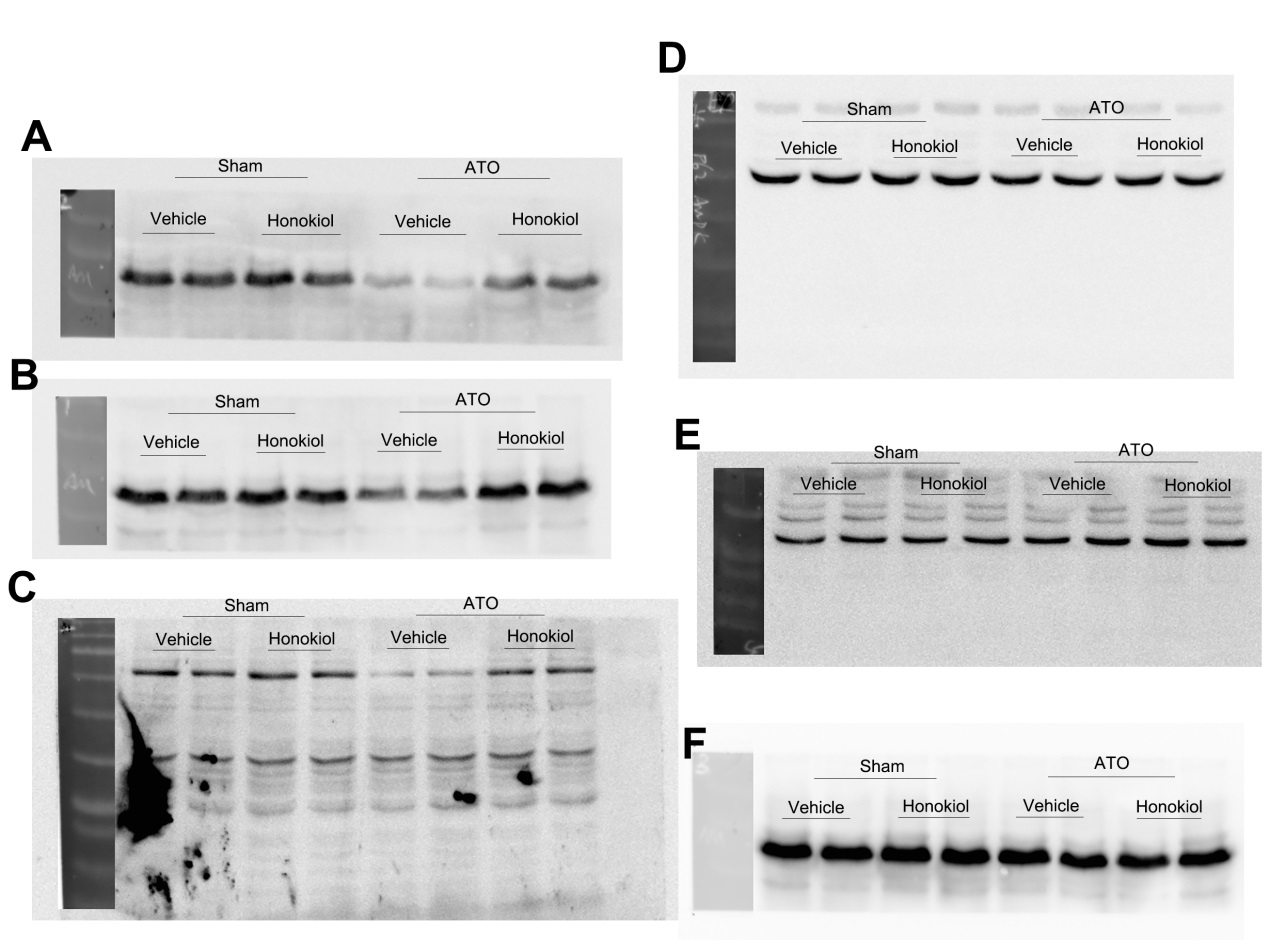


Supplementary figure 7

The expression levels of SIRT3 (A, B, C) and GAPDH (D, E, F). B and F were used as representative graphs.


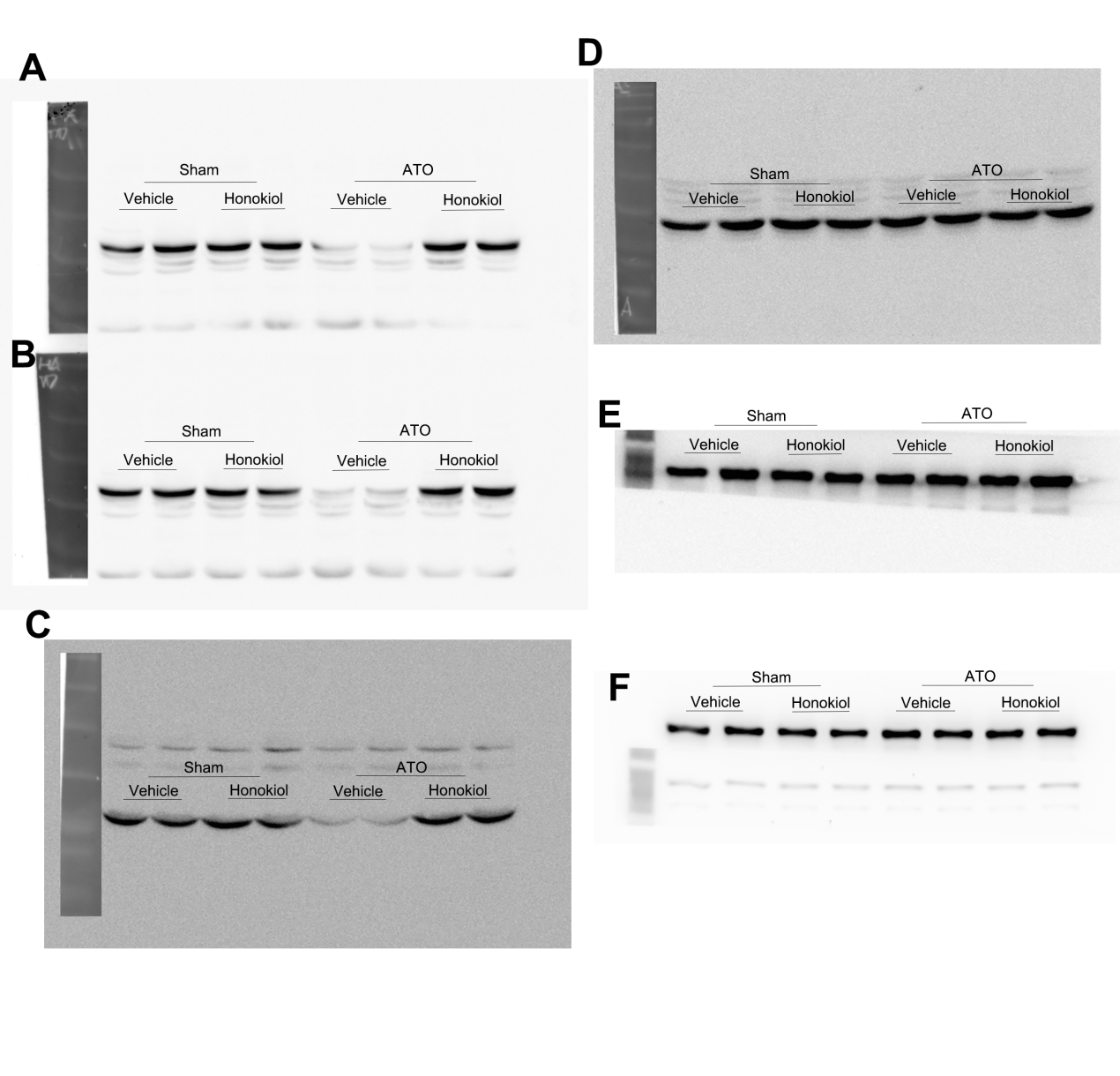

Supplement: Supplementary file 1 — Figure S1–S7 [file PRP2-10-e00914-s001.docx]
